# Reliability and Structural Validity of the Movement Assessment Battery for Children-2 in Croatian Preschool Children

**DOI:** 10.3390/sports7120248

**Published:** 2019-12-11

**Authors:** Ivan Serbetar, Jan Morten Loftesnes, Asgeir Mamen

**Affiliations:** 1 Department of Kinesiology, Faculty of Teacher Education, University of Zagreb, 10000 Zagreb, Croatia; 2 Faculty of Education, Arts and Sports, Western Norway University of Applied Sciences, 6856 Sogndal, Norway; jan.loftesnes@hvl.no; 3 School of Health Sciences, Kristiania University College, 0107 Oslo, Norway

**Keywords:** MABC-2, preschool children, reliability, structural validity, motor competence

## Abstract

Monitoring and assessment of the development of motor skills is an important goal for practitioners in many disciplines as well as researchers interested in motor development. A well-established tool for such purpose is the *Movement Assessment Battery for Children Second Edition* (MABC-2) which covers three age ranges and contains eight motor items in each range related to the manual dexterity, aiming and catching, and balance. The main aim of the study was to investigate the reliability and validity of the MABC-2 age band one in a sample of Croatian preschool children. Structural validity was assessed using confirmatory factor analysis (CFA). Measures of *relative* and *absolute* reliability were established by computing the intraclass correlation coefficients (ICC), standard error of the measurement (SEM), and smallest detectable change (SDC). About 17% of the children of the total sample fall into the categories of motor impairment and risk for impairment, respectively, while 83% were found to be in the category of normally developing children. Intraclass correlation coefficient for the total standard score was 0.79 while individual items, all except one, ranged from 0.70 to 0.83. *Drawing trail,* but also *throwing beanbag* and *one-leg balance* items presented large SEM and SDC values. CFA initially yielded a model with questionable fit to the data. After re-specification, excellent model fit was attained confirming the proposed three-factor model. Satorra–Bentler χ^2^(26) reached 38.56 (*p* = 0.054), *root mean square error of approximation* (RMSEA) was 0.028, *non-normed fit index* (NNFI) was 0.98, *adjusted goodness of fit* (AGFI) was 0.97, and *standardized root mean residual* (SRMR) was 0.030. All the variables loaded significantly, and only two significant standardized residuals have been found. Correlations between the factors were weak, supporting discriminant validity of the test. We found MABC-2 to be an appropriate instrument to assess the development of motor competences of preschool children.

## 1. Introduction

Motor competence or “the acquisition and refinement of skillful performance in a variety of movement activities” [1] (p. 158) is thought to be an important aspect of children’s engagement and disengagement, not only in physical activity [2] but also in children’s social and academic development [3]. Lack of children’s social cooperation could possibly result in emotional difficulties, poor social skills, lower academic achievement [4], reduced success within peer groups, or even experience of anxiety and depression [5]. Therefore, monitoring and assessment of the development of motor skills is an important goal both for practitioners in many disciplines and researchers interested in motor development.

A well-established tool for such purpose is the Movement Assessment Battery for Children - Second Edition (MABC-2) [6]. MABC-2 covers three age ranges and contains eight motor items in each range. Items are related to manual dexterity, aiming and catching, and balance. Separate standardized scores with adequate percentile values can be derived for each item, for each part of the test, and for the total score.

Following recent theoretical approaches in reliability studies, measures of absolute and relative reliability [7,8,9] must be established. Relative reliability may be assessed by quantifying correlation between repeated measures, usually by obtaining the intraclass correlation (ICC) [10]. Absolute reliability, on the other hand, refers to the variability of the scores from trial to trial (within subject/measurement), and it is not sample-dependent because the range of individual scores is not taken in account. The common estimate of absolute variability is the standard error of measurement (SEM), which is the measure of within-subject variation regarded as a “random variation in a measure when individual is tested many times” [7] (p. 2). An additional statistic called smallest detectable change (SDC, sometimes also called MDC—minimal detectable change) is increasingly being used as a benchmark for the interpretation of changes in scores. SDC indicates the smallest amount of change in score, which is due to a real change in score and not due to error in measurement. Considering that SDC is based on SEM, it is also expressed in original units of measurement with a confidence of 90% calculated from 1.65 × √2 × SEM and 95%, calculated from 1.96 × √2 × SEM.

The above-described approach to reliability has already been adopted in at least two MABC-2 psychometric studies [11,12]. Structural validity is also an important issue in test evaluation. Since MABC-2 was published in 2007, to our knowledge, only two other studies have considered factor structure of age band one, which were evaluated in a normative sample of 431 British children [13] and a sample of 183 Greek children [14]. Apart from establishing the factor structure, several other psychometric studies have been conducted, though only a few of them have been concerned with preschool children, and only two of those evaluated all four age groups in age band one separately [13,15].

Additionally, the current study is also concerned with the issue of test evaluation, in a particular national and cultural context, already raised by Brown and Lalor [16]. The aim of the present study is to assess reliability and validity of MABC-2 age band one, for the sample of Croatian preschool children.

## 2. Materials and Methods

### 2.1. Participants

Participants were 683 children (366 boys and 317 girls) aged 3 to 6 yrs. who attended kindergartens in North-West Croatia. The assessment was conducted during 2017. The data, showing the sample subdivided into age groups, are presented in Table 1. Only children without known health issues were included in the sample, and written informed consent was obtained from the children’s parents. All of the children were assessed individually according to the test rules. 

For various reasons (mostly refusal or failed items), 33 children did not complete the whole test, but their completed individual items were used in descriptive statistics. However, since the total test score cannot be obtained unless all of the tasks are done, only the subjects who completed all of the tests (*n* = 650) were included in the reliability and validity analyses.

### 2.2. Ethical Considerations

The parents or guardians of the children signed informed consent before the study began. It was clearly stated there that the child participated of free will and could at any time withdraw from the participation without giving a reason for doing so. It also stated that the anonymity was guaranteed and that the data would be protected. The subjects were treated according to the Helsinki Declaration, paying special attention to the paragraph for Vulnerable groups and individuals (§19–20).

### 2.3. Instrument

MABC-2 age band one is comprised of eight motor tasks which belong to three domains of motor performance: manual dexterity, aiming and catching, and balance. Manual dexterity contains three tasks: drawing trail, posting coins, and threading beads. The first task is scored by the number of errors the subjects make, while in the latter two are scored as the time in seconds taken to complete. Aiming and catching consists of throwing and catching the bean bag, which are both scored by the number of successful attempts. Balance includes one-leg balance which is scored as the time recorded, and walking heels raised and jumping on the mats which is scored as the number of correct attempts registered.

For two items—threading beads and one-leg balance—the testing included preferred and non-preferred hand and leg, respectively, and consequently ten raw scores and total test scores were obtained.

### 2.4. Procedure (Assessment)

The test protocol was translated into Croatian in order to standardize instructions for children and to enhance consistency in scoring among raters. Children were assessed individually in kindergartens in quiet and isolated rooms exactly according to the directions provided in the manuals. The data collection was done in 2017.

During the first assessment, four raters independently rated 36 children, thus providing the data for inter-rater reliability. Retest reliability was established by assessing 183 children repeatedly, in a period of 12–16 days after the first evaluation.

### 2.5. Data Analysis

Descriptive statistics parameters were calculated for the whole sample but also for the subsamples divided according to age. Based on recommendations as described in the introduction, in reliability studies more than one statistic should be obtained [7,8,9]. Therefore, intraclass correlation coefficients (ICC), standard error of measurement (SEM), and smallest detectable change (SDC) were calculated. SEM (as an estimate of absolute reliability) indicated the expected error in the measurement of an individual’s score expressed in real units of measurement, while SDC reflected the interval of confidence around an error.

For ICC calculation we adopted the ICC form described in Shrout and Fleiss [10] as ICC_2,1_, or two-way with random effect for absolute agreement. This is expressed in de Vet et al. [17] as: ICC_agreement_ = s^2^_between subj_./s^2^_between subj_.+ s^2^_trials_ + s^2^_residual_.

SEM is typically estimated by multiplying standard deviation by √1-ICC, but that form of ICC could substantially affect the result [8]. In addition, [7] it is also recommended that the error term from a two-way model should be employed because the one-way model combines random and systematic error. The above strategy is adopted in the present research, and SEM is calculated as stated in de Vet et al. [17] as SEM_agreement_ = √s^2^_trials_ − s^2^_residual_. Because of the different metrics of the MABC-2 items, SEM was also expressed as the percentage of the mean: SEM = (SEM/Mean) × 100. 

For the subsamples assembled on the basis of age, all reliability parameters were calculated using raw scores, because we assumed that it is more meaningful to obtain SEM and SDC values in the real unit of measurement than in standard scores.

In order to check the internal structure of the three-factor model proposed by Henderson et al. [6], confirmatory factor analysis was performed in LISREL 8.8 [18]. Due to the intention to directly compare the results, the same fit indexes used in the validity study of the authors of the test [13] were chosen.

We used Satorra–Bentler chi-square because data were not normally distributed (Mardia’s kappa = 116.0). In concordance with the aforementioned validity study of Schulz et al. [13], we also used root mean square error of approximation (RMSEA), the non-normed fit index (NNFI), the adjusted goodness of fit (AGFI), and the standardized root mean residual (SRMR). Criteria for the acceptance of the fit indexes were based on the relevant references from the field of structural equation modeling.

## 3. Results

### 3.1. Descriptive and Classification Results

Raw scores obtained in individual MABC-2 tasks are shown in Table 2. The results are not directly comparable between each age group because of the differences between performing and/or scoring of particular items for different ages.

While checking the frequencies, we observed that 60% of the children obtained the maximum score in *walking heels raised,* and 75% performed *jumping on the mats* with no error. In the subsample of 5 and 6-year-olds the percentage of maximal results in a *jumping* task was even higher with 84% of the children reaching the maximum, thus pointing to the ceiling effect.

Henderson et al. [6] provided categorization of children according to the traffic light system. In the proposed classification, which was based on percentile scores obtained on a normative sample of British children, children below 5th percentile (red category) are considered as motor impaired, between 5th and 15th percentile (yellow category) are children at risk for impairment and above 15th percentile are normally developing children. Table 3 shows the classification of the present study sample, in which only the children without failed items were considered. About 17% of the children of the total sample fall into the categories of motor impairment and risk for impairment, respectively, while 83% were found to be in the category of normally developing children.

### 3.2. Reliability

Although 183 children were retested, the children who refused to perform on the retest (*n* = 1), or those who had fallen into the “red“ category on one test occasion and in the “green“ category on the other test occasion (*n* = 9) were excluded from the analysis. We presumed that such a shift from one category to another was a motivational and not ability issue.

Total sample intraclass correlation coefficients based on the standard scores for individual tasks (Table 4) all ranked, except for one (jumping on mats), between 0.70 and 0.83, while ICC for the total standard score was found to be 0.79. Based on Koo and Li’s [19] suggestion, ICC values less than 0.5, between 0.5 and 0.75, between 0.75 and 0.90, and greater than 0.90 are indicative of poor, moderate, good, and excellent reliability, respectively.

Intraclass coefficients for each task and for total test score for single age groups were calculated on raw scores and their ranges across the items were somewhat wider (Table 4). ICC for the total test score for 3-year-olds was 0.53, while ICC for other age groups ranged from 0.75 to 0.85. Interestingly, almost all ICC values of different age groups for fine motor tasks were above 0.70, some of them even above 0.80, while for gross motor tasks some ICC values (catching and throwing) were found to be below 0.60 or even 0.50, respectively (Table 4). Similar moderate values were obtained for some balance tasks.

Considering that ICC is highly sample-dependent, we also calculated two sample-independent measures, standard error of measurement (SEM) and the smallest detectable change (SDC). Since we used raw scores, SEM and SDC are expressed in units of original measures. It should be noted that SEM rises as the value of measure rises, however, in the current research, both measures showed a large range of values because the metrics of MABC-2 tasks differ between items. To make the SEM values comparable between the items, they were also expressed relatively as a percent of the mean (SEM%). As shown in Table 5, drawing a trail had the largest SEM% in all age groups, but it is accompanied with one-leg balance items, and by throwing a beanbag. Accordingly, SDC measures were also higher in those tasks.

During the first assessment, four raters independently rated 36 children (Table 6). ICC values of 0.88 and 0.86 were obtained for drawing trail and catching beanbag, respectively, while all other values were 0.94 or higher. Using the total test score as an example, the SEM was 3.16 which is relatively low. SDC95 for the total score was 8.75, meaning that the change in the total score larger than the stated value is needed to ensure 95% certainty that the change in score is not due to the variability or measurement error of the tester, but rather a real change in score.

### 3.3. Confirmatory Factor Analysis (CFA)

**Figure 1:**
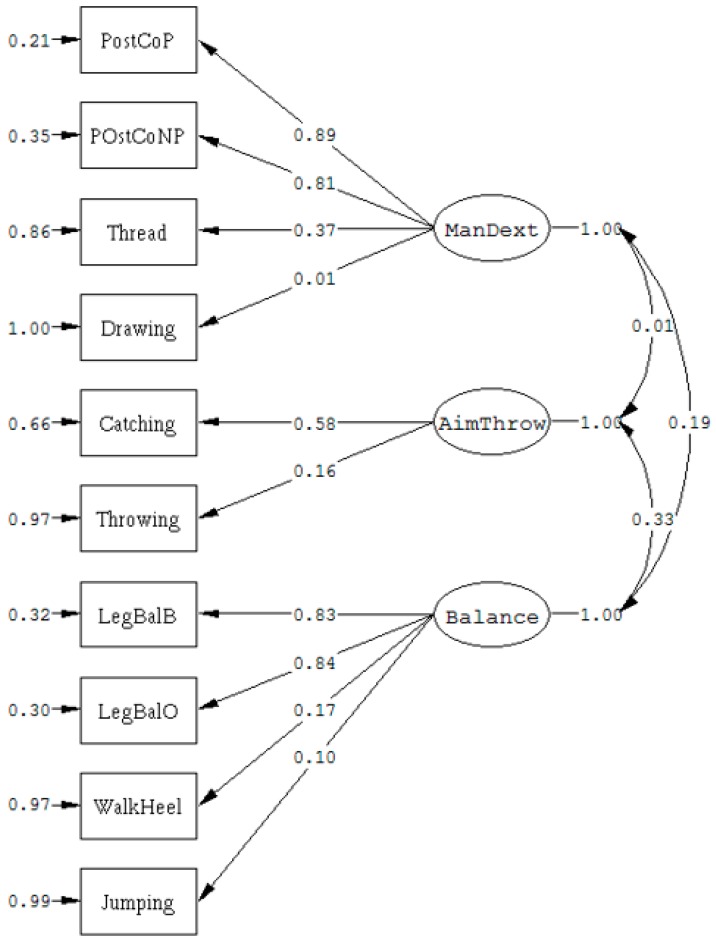
Path diagram of MABC-2.

The hypothesized set of relations in the model is shown on the path diagram (Figure 1).

The initial model was of a simple structure thus not allowing double loadings. At the beginning of confirmatory factor analysis (CFA), we checked for outliers and 44 cases (6.62%) with a significant *p*-value (*p* < 0.05) for Mahalanobis d-square were found and subsequently removed from data, which left 606 cases in the data set available for confirmatory factor analysis. According to Kline [20], the remaining sample may be apprehended as sufficiently large regarding either the rule of thumb, based on which “minimum sample size should be no less than 200 (preferably no less than 400, especially when observed variables are not multivariate normally distributed; p. 111) or 5–20 times the number of parameters to be estimated, whichever is larger” (p. 178). 

The multivariate distribution was also assessed and Mardia’s kappa of 116.10 was obtained with the standardized value of −3.03 (*p* = 0.002). As suggested by Byrne [21], the kappa values greater than 30 imply significant departure from multivariate normality, therefore estimation was achieved using the robust maximum likelihood method with the Satorra–Bentler scaled chi-square (S-Bχ^2^).

In the initial model (Figure 1) S-Bχ^2^(32) yielded 106.93 (*p* < 0.001), while the root mean square error of approximation with 90% confidence interval (RMSEA (90% CI)) was found to be 0.062 (CI 0.049–0.075). The non-normed fit index (NNFI) reached a value of 0.882, while adjusted goodness of fit index (AGFI) was 0.944, and standardized root mean square residual (SRMR) was 0.052.

Although some indices of fit were acceptable, we found that only half of the items loaded significantly on their respective factors, and that there were many large standardized residuals. Both indicators of dynamic balance loaded very little on their latent factors and showed large error variance not accounted for by their latent construct. Moreover, drawing seems not to be related to the manual dexterity factor in this particular sample.

After re-specification, which included general motor factor in the model, and by allowing three error terms to correlate, model fit improved substantially. The re-specified model yielded S-B χ^2^(26) of 38.56 (*p* = 0.054), RMSEA was 0.028 (<0.05 preferred; C.I. = 0.0|0.046), NNFI was 0.98 (≥0.95 preferred), AGFI was 0.97 (>0.90 preferred), and SRMR was 0.030 (<0.05 well fit). All of the variables loaded significantly on their respective factors, with the exception of drawing and jumping, which loaded directly onto the general motor factor. Only two standardized residuals larger than 2 (2.88, 2.40) were found.

The correlations between the factors of aiming and catching and balance (*r* = 0.33), and manual dexterity and balance (*r* = 0.19) were moderate and small, respectively, while manual dexterity and aiming and catching were not correlated (*r* = 0.01).

Correlations (Table 7), using standard item scores and total standard score were computed. All the items were significantly correlated with the total standard score ranging from *r* = 0.40 to *r* = 0.56 at *p* < 0.01. Most of the inter-item correlations were also significant ranging from 0.10 up to 0.69.

## 4. Discussion

The aim of the present study was to investigate the psychometric properties of the MABC-2 age band one in a sample of Croatian preschool children aged 3 to 6 years.

In terms of categorization of children related to the motor development, we found the ratio of the normally developing children and children at risk or motor impaired children to be relatively similar as in the Greek study [22], where 88% of the children were placed in the normally developing category, while 6.3% and 5.4% were placed in the risk for impairment and impairment categories, respectively.

On the contrary, in a Brazilian study [15], a somewhat lower percentage of children in the normally developing category was identified. They found 66%, 60%, 69%, and 88% of normally developing children in the age of 3, 4, 5, and 6 years, respectively. On the descriptive level of our data, we also found that more than half of the children obtained highest scores on balance tasks. That could be a scoring issue of the scale, but also an issue of intercultural differences [15,16,23].

Confirmatory factor analysis (CFA) was carried out to prove the three-factor structure of the original motor domains conceptualization of Henderson et al. [6]. Since the fit of our hypothesized model to the data was rather questionable, model re-specification was conducted. An approach from the study by Schulz et al. [13] was adopted, which included introducing general motor factor to the model. After several modifications of the model, excellent model fit was attained. Only drawing and jumping did not load on their respective factors, but they both loaded significantly on the general motor factor. All indices of fit were very close to those reported by Schulz et al. [13]. Apart from that study, the work conducted by Ellinoudis et al. [14] was the only one available for comparison. In their study, clear factor structure and higher loadings were reported. Yet, their sample was rather small (*N* = 183), except for chi-square, they reported only two indices of fit, and they did not provide information about residual variance.

Correlations between the factors in the present study were weak, which supported discriminant validity of the test. On the other hand, significant correlations found between items and total test score further confirmed the validity of the test. Inter-tester reliability was found to be excellent, yielding very high consistency among raters.

Retest reliability was calculated using raw scores for individual items because SEM and SDC are more meaningful when expressed in natural units of measurement than in standard scores. Considering the total sample, retest reliability expressed in terms of ICC was more than acceptable with only one item positioned below 0.70. The total standard score ICC was somewhat lower than the levels of reliability obtained in the study by Ellinoudis et al. [14], who reported ICC for the total score of 0.85, while individual items ranged from 0.66 to 0.96. Nonetheless, when compared to the Holm et al. study [11], where raw scores were also used, ICC’s were higher. Unfortunately, values of items’ ICCs were not stated by the authors of those studies, thus making the comparison with current values impossible. 

When the sample in the present study was divided into age groups, the range of ICC coefficients became rather less narrow. However, when comparing the total test scores’ ICCs between age groups in our study, ICC of 0.53 obtained for 3-year-olds, was the only one which was unsatisfactory. Smits-Engelsman et al. [12] obtained a greater ICC coefficient (0.94) for the same age, but individual item scores’ ICCs varied in their study as well, namely between 0.67 and 0.85. They also argued that some MABC-2 tasks were more challenging for 3-year-olds than for older children. It may also be presumed that social unfamiliarity of the children with the raters also had certain impact on the motivation of those children for maximal performance and concentration. Thus, changes over time may be more pronounced in the youngest group than in older preschool children.

Nevertheless, from the view of psychometric theory, both high and low ICC values should be taken with caution, because, as stated in Weir [8] “large ICC can mask poor trial-to-trial consistency when between subjects variability is high” and “conversely, a low ICC can be found even when trial-to-trial variability is low if the between subjects variability is low” (p. 237). Weir [8] also pointed out the importance of the source of the error which should be briefly addressed. Namely, error term in ANOVA expresses the interaction of the subjects and trials, where a small error may reflect that scores change similarly in the repeated trials which may result in a significant trial effect, meaning that there is some systematic error present. On the contrary, random error may exist in data when changes between trials are not consistent (some scores fall, some rise). Since a two-way model allows the error to be partitioned, we checked the mean squares in ANOVA output, finding significant trial effect for total test score, throwing the ball, drawing, threading beads, posting coins non-preferred hand, jumping on the mats, and leg balance on the non-preferred leg. This indicates the possible systematic error, caused most likely by the familiarization of the children with the test, known also as “learning effect”.

In some of the remaining items, examination of SEM and SDC indicated high random error. Large values of SEM and SDC have been found in all age groups for drawing, one-leg balance and throwing, while aiming and catching seems to be problematic for 3 and 4-year-olds and jumping on the mats for 5-year-olds.

Drawing presented the highest measurement error in our study, and it was also the item with the lowest ICC in the studies by Ellinoudis et al. [14] and in Smits-Engelsman et al. [12]. Moreover, drawing has also shown validity issues in the study by Schulz et al. [13], as well as in the present study, which may suggest that the drawing in the preschool age is more related to some other abilities (i.e., visual motor integration) than to fine motor skills alone.

Findings of the present study showed acceptable overall evidence of validity and reliability of the MABC-2 for age band one, suggesting that it can be a useful tool to assess motor competences in Croatian preschool children.

## 5. Conclusions

We found MABC-2 to be an appropriate instrument to assess the development of motor competences of preschool children. MABC-2 tasks are intuitive and easy to perform for the children, and the test could differentiate the level of attained motor skills. Dilemmas about the possible cultural limitation of MABC-2 raised here, but also in other studies, should be further investigated.

## Figures and Tables

**Table 1:** Demographic characteristics of participants.

Gender	3 yrs.	4 yrs.	5 yrs.	6 yrs.	Total
boys	33	65	141	127	366
girls	37	68	118	94	317
total	70	133	259	221	683

**Table 2:** Raw scores of individual MABC-2 items.

Mean (SD)								
MABC-2 Item	3 yr. (*n* = 70)	*n*	4 yr. (*n* = 133)	*n*	5 yr. (*n* = 259)	*n*	6 yr. (*n* = 221)	*n*
Posting coins pref. hand	14.13 (4.03)	70	14.62 (6.66)	133	20.61 (4.46)	259	18.62 (3.08)	221
Posting coins non-pref. hand	16.19 (4.37)	70	15.77 (7.66)	131	22.96 (5.91)	259	19.99 (3.94)	219
Threading beads	65.33 (30.2)	68	55.34 (27.61)	128	74.2 (39.29)	250	60.76 (24.42)	218
Drawing trail	4.95 (3.18)	67	2.98 (2.71)	133	2.36 (2.50)	255	1.83 (2.27)	221
Catching beanbag	5.05 (2.15)	70	6.81 (2.19)	132	7.68 (1.98)	259	8.49 (1.59)	221
Throwing beanbag on to mat	4.02 (1.94)	69	4.65 (1.82)	132	5.2 (2.08)	259	5.56 (2.08)	221
One-leg balance pref. leg	6.02 (4.51)	68	10.24 (7.54)	131	19.41 (9.44)	257	24.22 (8.11)	221
One-leg balance non-pref. leg	4.55 (3.19)	68	9.26 (7.15)	131	16.47 (9.79)	259	21.26 (9.3)	220
Walking heels raised	10.20 (4.48)	68	12.36 (3.86)	133	13.28 (2.99)	257	13.43 (2.78)	221
Jumping on mats	3.84 (1.18)	69	4.41 (1.01)	133	4.66 (0.86)	258	4.77 (0.79)	221
Total test score	79.71 (12.27)		72.08 (10.76)	-	74.51 (12.68)	-	71.16 (12.45)	-

**Table 3:** Mean percentile values and traffic light system classification.

	Percentile	Classification *n* (%)		
Age	Mean (SD)	Motor Impairment Red Category	At Risk Yellow Category	Normal Development Green Category
3 yr. (*n* = 62)	51.53 (26.80)	2 (3)	3 (5)	57 (92)
4 yr. (*n* = 125)	35.02 (23.36)	10 (8)	8 (6)	107 (86)
5 yr. (*n* = 246)	41.04 (26.20)	23 (9)	14 (6)	209 (85)
6 yr. (*n* = 217)	34.49 (25.74)	23 (11)	29 (13)	165 (76)
Total (*N* = 650)	38.70 (26.03)	58 (9)	54 (8)	538 (83)

**Table 4:** Intraclass correlation coefficients (ICC).

	ICC (±95% CI)				
	Based on Raw Scores			Based on Standard Scores	
MABC-2 Item	3 yr. (*n* = 25)	4 yr. (*n* = 27)	5 yr. (*n* = 53)	6 yr. (*n* = 68)	Total (*N* = 173)
Posting coins pref. hand	0.82 (0.60 0.92)	0.69 (0.33 0.86)	0.74 (0.55 0.85)	0.85 (0.76 0.91)	0.76 (0.68 0.82)
Posting coins non- pref. hand	0.84 (0.64 0.93)	0.68 (0.28 0.85)	0.83 (0.70 0.91)	0.72 (0.51 0.83)	0.72 (0.62 0.79)
Threading beads	0.75 (0.45 0.89)	0.92 (0.80 0.97)	0.88 (0.75 0.94)	0.70 (0.48 0.82)	0.80 (0.71 0.86)
Drawing trail	0.78 (0.50 0.91)	0.84 (0.66 0.93)	0.81 (0.67 0.90)	0.79 (0.66 0.87)	0.83 (0.78 0.88)
Catching beanbag	0.55 (0.05 0.80)	0.92 (0.83 0.97)	0.65 (0.40 0.80)	0.48 (0.16 0.68)	0.70 (0.60 0.78)
Throwing beanbag on to mat	0.66 (0.22 0.85)	0.67 (0.29 0.85)	0.57 (0.26 0.75)	0.78 (0.63 0.86)	0.75 (0.66 0.81)
One-leg balance pref. leg	0.84 (0.64 0.93)	0.68 (0.28 0.85)	0.80 (0.66 0.89)	0.49 (0.19 0.68)	0.81 (0.75 0.86)
One-leg balance non-pref. leg	0.80 (0.55 0.91)	0.62 (0.16 0.82)	0.77 (0.60 0.87)	0.68 (0.46 0.80)	0.77 (0.69 0.83)
Walking heels raised	0.87 (0.70 0.94)	0.88 (0.73 0.94)	0.83 (0.70 0.90)	0.64 (0.42 0.78)	0.78 (0.70 0.83)
Jumping on mats	0.92 (0.82 0.96)	0.78 (0.52 0.90)	0.59 (0.30 0.76)	0.66 (0.45 0.79)	0.66 (0.54 0.75)
TTS/TSS *	0.53 (0.10 0.80)	0.85 (0.64 0.94)	0.75 (0.54 0.86)	0.83 (0.62 0.91)	0.79 (0.71 0.85)

Note: * TTS -*total test score* calculated for age groups and based on raw scores/TSS *total standard score* calculated for the total sample.

**Table 5:** Standard error of measurement (SEM) and smallest detectable change (SDC) values for each age.

Age	Reliability Parameters	Posting Coins Pref. Hand	Posting Coins Non-Pref. Hand	Threading Beads	Drawing Trail	Catching Beanbag	Throwing Beanbag on to Mat	One-Leg Balance Pref. Leg	One-Leg Balance Non-Pref. Leg	Walking Heels Raised	Jumping on Mats	TTS
3 yr.	SEM	1.23	1.37	9.93	1.76	1.87	1.13	1.25	0.95	2.20	0.32	8.40
	SEM%	8.16	8.39	17.74	26.96	36.68	25.95	28.08	31.66	20.75	7.65	10.64
	SDC90	2.86	3.19	23.14	4.11	4.36	2.64	2.92	2.22	5.13	0.74	19.58
	SDC95	3.41	3.80	27.52	4.88	5.18	3.13	3.47	2.64	6.09	0.88	23.27
4 yr.	SEM	3.01	3.39	4.90	1.09	0.84	1.30	4.56	4.44	2.34	0.43	5.30
	SEM%	23.34	24.17	12.02	41.39	12.17	26.66	52.31	58.17	20.39	9.14	7.19
	SDC90	7.01	7.89	11.42	2.54	1.95	3.02	10.63	10.34	5.45	1.00	12.34
	SDC95	8.33	9.38	13.57	3.02	2.32	3.60	12.63	12.29	6.49	1.19	14.67
5 yr.	SEM	2.24	1.91	8.78	1.29	1.00	1.41	7.72	6.17	1.75	1.53	6.84
	SEM%	11.82	8.86	16.21	68.12	12.20	26.27	35.83	38.25	12.72	31.35	8.48
	SDC90	5.22	4.44	20.45	3.00	2.32	3.29	17.99	14.38	4.09	3.56	15.93
	SDC95	6.20	5.28	24.31	3.57	2.76	3.91	21.39	17.09	4.86	4.23	18.94
6 yr.	SEM	0.95	1.83	10.65	1.12	0.97	1.14	10.22	6.57	1.78	0.12	5.77
	SEM%	5.23	9.23	20.31	68.98	10.67	21.84	41.52	38.02	11.48	2.4	7.63
	SDC90	2.21	4.27	24.81	2.62	2.26	2.65	23.82	15.31	4.15	0.28	13.45
	SDC95	2.62	5.08	29.49	3.11	2.69	3.15	28.32	18.2	4.93	0.33	15.99

**Table 6:** Inter-rater reliability.

	Posting Coins Pref. Hand	Posting Coins Non-Pref. Hand	Threading Beads	Drawing Trail	Catching Beanbag	Throwing Beanbag on to Mat	One-Leg Balance Pref. Leg	One-Leg Balance Pref. Leg	Walking Heels Raised	Jumping on Mats	TTS
ICC (±95% CI)	0.96 (0.91–0.98)	0.97 (0.94–099)	0.99 (0.99–1.00)	0.88 (0.73–095)	0.86 (0.68–0.94)	0.95 (0.88–0.98)	0.99 (0.97–0.99)	0.97 (0.92–0.99)	0.99 (0.98–1.00)	0.95 (0.88–0.98)	0.94 (0.86–0.97)
SEM	1.12	0.96	0.59	1.01	0.67	0.68	1.73	2.21	0.14	0.24	3.16
SDC95	3.10	2.67	1.65	2.80	1.84	1.88	4.79	6.13	0.38	0.67	8.75
SDC90	2.61	2.25	1.38	2.35	1.55	1.58	4.03	5.15	0.32	0.56	7.36

**Table 7:** Correlations between items and total test score.

MABC-2 Item	PostCoP	PostCoNp	Thread	Drawing	Catching	Throwing	LegBalB	LegBalO	WalkHeel	Jumping
PostCoNp	0.69 **									
Thread	0.31 **	0.28 **								
Drawing	−0.01	0.00	0.08 *							
Catching	0.05	0.01	−0.05	0.08 *						
Throwing	−0.03	−0.02	0.10 *	0.19 **	0.12 **					
LegBalB	0.15 **	0.13 **	0.10 *	0.02	0.16 **	0.06				
LegBalO	0.12 **	0.11 **	0.14 **	0.03	0.13 **	0.04	0.64 **			
WalkHeel	0.03	0.01	0.05	0.11 **	0.10 *	−0.05	0.19 **	0.19 **		
Jumping	0.10 *	00.05	0.11 **	0.13 **	0.18 **	0.08 *	0.12 **	0.06	0.06	
TSS	0.43 **	0.41 **	0.56 **	0.49 **	0.40 **	0.40 **	0.42 **	0.41 **	0.40 **	0.41 **

**: significant at the 0.01 level; *: significant at the 0.05 level; PostCoP-Posting coins preffered hand; PostCoNp-Posting coins non-preffered hand; Thread-Threading beads; Draw–Drawing trail; Catching–Catching beanbag; Throwing–Throwing beanbag; LegBalB-Leg balance better leg; LegBalO-Leg balance other leg; WalkHeel-Walking heels raised; Jumping–Jumping on mats.
